# A*-TEB: An Improved A* Algorithm Based on the TEB Strategy for Multi-Robot Motion Planning

**DOI:** 10.3390/s25196117

**Published:** 2025-10-03

**Authors:** Xu Li, Tuanjie Li, Yan Zhang, Yulin Zhang, Ziang Li, Lixiang Ban, Kecheng Sun

**Affiliations:** 1 College of Information Engineering, Tarim University, Alar City 843300, China; lixu2866@126.com; 2 School of Mechano-Electronic Engineering, Xidian University, Xi’an 710071, China; marks.zhang@megarobo.tech (Y.Z.); 22041110074@stu.xidian.edu.cn (Y.Z.); 23041212563@stu.xidian.edu.cn (Z.L.); 24041212487@stu.xidian.edu.cn (L.B.); 24041212660@stu.xidian.edu.cn (K.S.)

**Keywords:** multi-robot systems, improved A* algorithm, Timed Elastic Band (TEB), integrated motion planning, autonomous navigation

## Abstract

Multi-robot motion planning (MRMP) requires each robot to possess strong local planning capabilities while maintaining global consistency. However, existing research often fails to address both global and local planning simultaneously, resulting in conflicts in real-time path execution. The A* algorithm is widely used for global path planning due to its adaptability and search efficiency, while the Timed Elastic Band (TEB) algorithm excels in local trajectory optimization and real-time dynamic obstacle avoidance. This paper presents a novel motion planning framework integrating an improved A* algorithm with an enhanced TEB strategy to address both levels of planning collaboratively. The proposed improvements include the introduction of steering costs and dynamic weights into the A* algorithm to enhance path smoothness and efficiency, and a hierarchical obstacle treatment in TEB for improved local avoidance. Simulation and real-world experiments conducted with ROS confirmed the feasibility and effectiveness of the method. Compared to the traditional A* algorithm, the proposed framework reduces the average path length by 5.2%, shortens completion time by 11.5%, and decreases inflection points by 66.7%, demonstrating superior performance for multi-robot systems in dynamic environments.

## 1. Introduction

An intelligent robot is a system with enhanced perception, decision-making, and effects that is capable of simulating human behavior, emotions, and thinking [1,2,3]. Research on intelligent robots spans multiple disciplines, including mechanics, electronics, control, artificial intelligence, computers, and sensors, and has become a global research hotspot [4,5,6]. With the advent of 5G communication technology, robotics is poised to replace information technology as a new economic growth engine. The problem of multi-robot cooperative motion planning is a significant challenge in the field of intelligent robot systems [7,8]. This problem involves enabling multiple robots to coordinate with each other during their movement, avoiding collisions and conflicts, and reaching target tasks optimally under specific constraints. It is considered a crucial technology for enhancing the operational efficiency of multi-robot systems. Current solutions for multi-robot cooperative motion planning can be classified into two categories based on the scope of planning: global planning methods and local planning methods.

In global planning methods, the planning process must consider all robots within the entire system, performing path planning and coordination on a global scale. Commonly used global planning methods include Dijkstra, the A* algorithm, the D* algorithm, and the RRT algorithm [9,10]. Compared with other algorithms, the A* algorithm combines cost functions with heuristic information, significantly improving search efficiency while ensuring path optimality, and exhibits advantages in efficiency, flexibility, and extensibility. Zhang et al. [11] designed a method based on rule formulation that uses an improved A* algorithm and a reservation table for multi-robot collaborative motion planning in intelligent warehouses. However, this method suffers from limited scalability. Milad et al. [12] proposed a method for continuous environments that employs an artificial potential field algorithm for initial path finding, followed by an improved genetic algorithm for optimal path selection. This approach considers path length, smoothness, and safety, but the algorithm is prone to falling into local optima. Zhang et al. [13] proposed an improved A* algorithm for AGV path planning that addresses issues related to excessive turning points and their proximity to obstacles. Their approach dynamically adjusts weights in the cost function based on turning angles and integrates the artificial potential field method to enhance the heuristic function with obstacle information. Lin et al. [14] developed an improved A* algorithm specifically designed for autonomous vehicle path planning in unstructured environments, incorporating redundant safety spaces to filter out impassable narrow roads and prevent collisions, while also introducing a prejudgment planning strategy and a redundant inflection point elimination technique to generate shorter and smoother paths. The improved only A* algorithm often results in limitations such as insufficient path smoothness and restricted global optimality in complex or dynamic environments. In contrast, integrating A* with other advanced algorithms can retain its efficient search capability while significantly enhancing path quality, adaptability, and environmental robustness, making it a more practical and effective strategy.

Fu et al. [15] proposed an improved A* algorithm for enhancing the path planning of indoor mobile robots. Their method involves modifications to the heuristic function and evaluation model to reduce turning points and angles. Additionally, they integrated a novel obstacle detection technique based on potential field theory to maintain safe distances from obstacles. Liao et al. [16] proposed an A* algorithm enhanced with Ant Colony Optimization (ACO). This approach combines A*’s initial path search with ACO to refine the paths, leading to reductions in path length and total turning angles, as well as improved trajectory smoothness. Luo et al. [17] combined an adaptive A* algorithm with an improved Dynamic Window Approach (DWA) for enhanced path planning. The adaptive A* algorithm optimizes global paths by utilizing dynamic weights and removing redundant points, while the improved DWA focuses on local path smoothing and obstacle avoidance. Experimental results demonstrated that this integrated approach leads to improved search efficiency, shorter paths, and better obstacle avoidance compared to traditional methods. These methods provide a better solution for the global planning of multi-robot cooperative motion planning, but there are some drawbacks, such as too many search nodes and the inability to achieve dynamic obstacle avoidance.

In local planning methods, each robot makes decisions based on its surrounding environment or nearby robots, dividing the overall problem into multiple local subproblems for processing. The commonly used local planning methods are the TEB algorithm, the artificial potential field method, particle swarm optimization, genetic algorithms, and ant colony algorithms [18,19]. Fox et al. [20] proposed the Dynamic Window Approach (DWA) based on the motion dynamics of the robot, enabling local obstacle avoidance. However, this method handles dynamic and static obstacle expansion similarly, resulting in suboptimal obstacle avoidance in certain scenarios. Li et al. [21] proposed an improved method for DWA that does not account for the size constraints of the robot. Yuting Liu et al. [22] further enhanced the DWA method by improving the evaluation function to address its shortcomings in random obstacle avoidance. Van et al. [23] introduced the Reciprocal Velocity Obstacle (RVO) method, which calculates bidirectional velocity obstacles between each robot and its neighbors, then adjusts velocities to avoid collisions. Bharath et al. [24] improved the RVO algorithm by incorporating chance constraints. Rösmann et al. [25] proposed the Timed Elastic Band (TEB) method, which introduces the concept of a Timed Elastic Band, allowing the robot to flexibly adjust the local path within a specific time range to avoid obstacles and conflicts. Zhang et al. [26] adapted TEB into a two-stage algorithm for the autonomous parking of driverless vehicles. Smith et al. [27] further improved the optimization efficiency of the TEB algorithm by introducing the concept of self-centered perceptual spatial navigation. Among the above methods, compared with other local planning algorithms, the TEB algorithm has certain advantages in real-time dynamic obstacle avoidance, and it is a better solution to solve the local planning in the collaborative motion planning of multiple robots, but the method has the shortcomings of large computation and easily falling into the local optimal solution. We conducted a comparative analysis of various improved A* algorithms and TEB variants, and the results are presented in Table 1.

However, there are still some limitations in the studies mentioned above. On the one hand, global planning algorithms are global and optimal; local planning algorithms are in real time. However, using global planning algorithms or local planning algorithms individually cannot guarantee the safety and stability of individual robots in a multi-robot system [27,28]. On the other hand, scholars have explored a variety of global planning methods or local planning methods for multi-robot systems, but there are fewer fusion methods that integrate global and local planning [29,30,31,32].

Therefore, given the limitations identified in previous studies, it is evident that neither global nor local planning algorithms alone can ensure the safety and stability of individual robots in a multi-robot system. To address this challenge, we propose a comprehensive motion planning approach that fuses global and local planning methods. Specifically, we integrate an improved A* algorithm with the Timed Elastic Band (TEB) strategy to enhance the effectiveness of cooperative motion planning in multi-robot systems. The key contributions of our work are summarized as follows:

1. A novel integrated motion planning framework combining an improved A* algorithm for global path planning and an enhanced TEB strategy for local trajectory optimization to ensure safety, consistency, and real-time performance in multi-robot systems. An improved A* algorithm by increasing the flexibility and adaptability of the A* algorithm in dynamic environments by incorporating steering costs and dynamic weights.
2. An improved A* algorithm with steering cost and dynamic weights to reduce inflection points and enhance search efficiency in dynamic environments.
3. An improved TEB algorithm that simplifies irregular obstacle processing through equivalent circular modeling and a hierarchical planning strategy (safety layer, intermediate layer, and collision layer) for robust dynamic obstacle avoidance.
4. Comprehensive validation through ROS-based simulations and real-world experiments involving heterogeneous mobile robots, demonstrating the feasibility and scalability of the proposed approach.

The organization of this paper is as follows: Section 2 provides a review of the principles of the traditional A* algorithm and the TEB algorithm, and an analysis of the advantages and problems associated with each. In Section 3, we propose a hybrid motion planning method that improves upon the A* algorithm and the TEB algorithm, and a multi-robot motion planner is constructed based on the move_base framework, which employs the aforementioned multi-robot motion planning method. In Section 4, simulations are conducted using ROS (Robot Operating System) Melodic, Gazebo7, and Rviz1.0, and the proposed approach is evaluated. Moreover, experiments were conducted in a real-world setting with five heterogeneous mobile robots to assess the viability of the proposed collaborative motion planning approach. In the final section, we present a discussion of related work and offer a brief conclusion.

## 2. Algorithmic Principle

This section reviews the principles of the traditional A* and TEB algorithms, highlighting their respective strengths and existing limitations to establish the basis for the proposed improvements.

### 2.1. Principles and Existing Problems of Traditional A* Algorithm

This subsection introduces the principles of the traditional A* algorithm and analyzes its existing problems to provide a foundation for subsequent algorithmic improvements.

#### 2.1.1. A* Algorithm Principle

**Figure 1:**
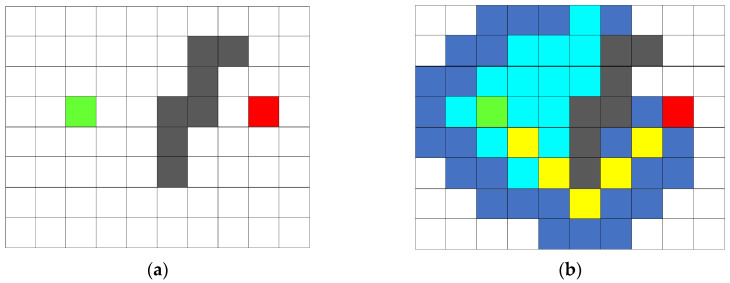
This is an A* algorithm search path diagram. The images are labeled alphabetically as (**a**) before searching and (**b**) search completed.

The A* algorithm has the characteristics of being applicable to a variety of environments, ease in finding optimal solutions in discrete spaces, and fast search [33]. The A* algorithm combines the completeness of the Dijkstra algorithm and the efficiency of the Breadth-First Search algorithm with the efficiency of greedy best-first search. The Dijkstra algorithm only considers the actual cost of the path that has been traveled, has no directionality in the path search process, and is less efficient; the A* algorithm uses a heuristic function to estimate the cost from the current node to the target node, combining the historical cost and future search based on the expected cost. The cost of the expandable nodes around the current node is calculated according to the cost function, and the node with the smallest cost is selected as the next node on the path. The above steps are repeated until the expanded node finds the target task point and finally obtains a path from the starting point to the target task point. The A* algorithm search path is shown in Figure 1. Green represents the starting point position of the agent, red represents the target task point position, black represents obstacles, the yellow path is the optimal path found, white represents the free grid, and the remaining color grids represent the nodes expanded by the A* algorithm search process.

The estimated cost of each node expanded by the A* algorithm is calculated from the historical cost and the future expected cost. The estimated cost function of the A* algorithm is shown in Equation (1):

(1) f(n)=g(n)+h(n)

where n represents the current node that the A* algorithm needs to expand and calculate the cost when searching for a path, f(n) represents the estimated cost of the current n node, g(n) represents the historical cost of the current node (that is, the agent starts from the starting point and reaches the current n node, which is the cost required), and h(n) represents the future expected cost of the current n nodes reaching the target task point, which is the heuristic function of the A* algorithm.

The historical cost calculation formula in the A* algorithm is shown in Equation (2):

(2) $$g(n)=\sqrt{{\left(x_{start}-x_{current}\right)}^{2}-{\left(y_{start}-y_{current}\right)}^{2}}$$

where $x_{start}$ represents the *x*-axis coordinate value of the starting point, $x_{current}$ represents the *x*-axis coordinate value of the current current node, $y_{start}$ represents the *y*-axis coordinate value of the starting point, and $y_{current}$ represents the *y*-axis coordinate value of the current current node.

Commonly used heuristic functions h(n) in the A* algorithm include Manhattan distance, Euclidean distance, and Chebyshev distance. Manhattan distance is suitable for agents that only need to move up, down, left, and right; Euclidean distance is suitable for robots moving in any direction; and Chebyshev distance is suitable for front, left-front, back, right-back, left, left-back, right, and right-front directions. For mobile robots, since the multi-robot collaborative motion planning problem studied in this article requires the robot to move in any direction to complete the target task, Euclidean distance is selected as the heuristic function of the A* algorithm.

#### 2.1.2. Problems with the A* Algorithm

Although the traditional A* algorithm can search for a better path for reachable target task points, there are also some problems that need to be improved in the search process of the traditional A* algorithm based on actual development conditions. This part first explains the problems existing in the search process of the traditional A* algorithm through the built raster map and analysis, and further explains what impact these problems will have on the planning of the agent, providing direction for the improvement of the next part.

**Figure 2:**
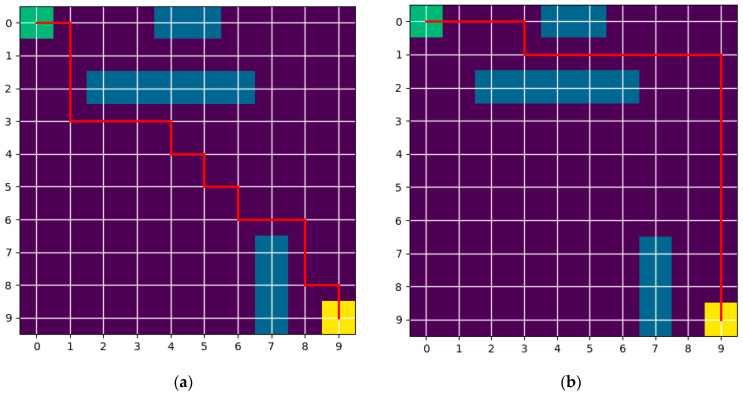
This is a schematic diagram of the A* algorithm planning results. The images are labeled alphabetically as (**a**) first path and (**b**) second path.

A 10 × 10 grid map is created. Green represents the starting point of the robot, yellow represents the robot’s target task point, blue represents the static obstacles existing in the grid map, and red represents the optimal path searched by the A* algorithm. As shown in Figure 2, the paths searched by the two A* algorithms are given. The path distances of the two results are equal to 18. However, it can be seen from Figure 2 that the first path has more inflection points than the second path. More points mean that the robot has more acceleration and deceleration processes during movement, and at the same time, it increases the corresponding control difficulty. In order to reach the target point, the corresponding movement time will also increase. Therefore, the first problem to be solved in this section is the problem of the traditional A* algorithm with many inflection points.

Compared with traditional blind search algorithms such as BFS and Dijkstra, the traditional A* algorithm improves search efficiency by adding a heuristic function. It adds constraints on the basis of Dijkstra’s algorithm to change its trend. Under the conditions of acceptability and monotonicity, the algorithm can also obtain the optimal solution. However, in the search process of the traditional A* algorithm, the expanded nodes are determined by the cost function. Therefore, in addition to the problem of multiple inflection points, in the path search process of the traditional A* algorithm, there are also a large number of nodes that are not related to the final path. It is time-consuming and reduces search efficiency. Through the above analysis, improving the search efficiency of the traditional A* algorithm is the second problem to be solved in the motion planning of this article. When using the traditional A* algorithm for path search, the inflection point of the generated path causes the path curvature to be insufficiently continuous, which is detrimental to the movement of most current intelligent entities (uncrewed ships, drones, uncrewed vehicles, etc.) and makes it difficult to meet actual motion requirements, which also increases the complexity of actually controlling the robot. Therefore, optimizing the path generated by the traditional A* algorithm is the third problem to be solved in motion planning in this article.

### 2.2. Principle of Traditional TEB Algorithm

This subsection explains the principles of the traditional TEB algorithm and discusses its limitations, which motivate the proposed enhancements in this paper.

The TEB algorithm is an improvement based on the Elastic Band (EB) algorithm [34]. The TEB algorithm introduces the concept of a time difference sequence to improve the EB algorithm. By introducing time intervals between consecutive poses, the TEB algorithm can better simulate the movement of an agent in a dynamic environment and avoid collisions with obstacles more effectively. Therefore, the TEB algorithm has higher accuracy and robustness in motion planning and trajectory optimization [35].

The key idea of the TEB algorithm is to realize the adjustment and integration of attitude and time differences by optimizing a weighted multi-objective function in real time. The weighted multi-objective function is as shown in Equation (3):

(3) $$f(B)=\sum_{k}\lambda_{k}f_{k}(B)$$

where f(B) represents the weighted multi-objective function, and $\lambda_{k}$ represents the weight value of the objective function $f_{k}(B)$. The objective functions $f_{k}(B)$ of the TEB algorithm can be categorized into two types: constraints on velocity and acceleration based on penalty functions, as well as trajectory-related objectives. The objective functions of the TEB algorithm include path point and obstacle functions, acceleration constraints and velocity constraints, and incomplete kinematics constraints and fastest path constraints.

The optimized TEB is expressed as Equation (4):

(4) $$B^{*}=\mathrm{argmin}f(B)$$

where $B^{*}$ represents the optimized TEB output path.

## 3. Algorithm Improvement and Integration

### 3.1. Improved A* Algorithm

To solve the problem of multiple turning points, the turning cost is introduced to improve the traditional A* algorithm. The turning cost is introduced to guide the traditional A* algorithm to generate straight paths and reduce turning points. The turning cost formula is shown in Equation (5):

(5) s(n)=angle(Current,parent(Current))

where angle(Current,parent(Current)) represents the angle between the current node Current and its parent node.

The angle can be calculated by vector operations. First, the vector and the module of the vector are calculated from the parent node to the current node, then the vector and the module of the vector are calculated from the current node to the neighbor node, and finally, the angle between the two vectors is calculated. The calculation formula is shown in Equation (6):

(6) $$angel=\cos^{-1}(\frac{dot(v_{1},v_{2})}{\left\|v_{1}\right\|\cdot \left\|v_{2}\right\|})$$

where $v_{1}$ and $v_{2}$ represent the vectors from the parent node to the current node and the current node to the neighbor node, respectively; dot(⋅) represents the dot product operation of the vector; and $\left\|v_{1}\right\|$ and $\left\|v_{2}\right\|$ represent the modules of the two vectors, respectively.

In order to solve the problem of multiple turning points, this paper introduces the concept of turning the cost into the estimated cost function, and Equation (1) is improved to Equation (7):

(7) f(n)=g(n)+h(n)+s(n)

In order to solve the problem of generating too many irrelevant nodes and reducing the search efficiency, dynamic weights are introduced into the future expected cost. The heuristic function of the traditional A* algorithm not only affects the search direction but also affects the search efficiency of the A* algorithm. The heuristic function acts as a guiding factor in the search process of the algorithm and accelerates the search by providing an optimistic estimate of the remaining cost to reach the goal, which is a process that focuses on exploring lower-cost paths. The introduction of dynamic weights makes the A* algorithm have a larger weight in the early stage of search, and it can search for nodes closer to the target task point faster. It can avoid premature convergence in the later stage of search and pay more attention to generating higher-quality and higher-accuracy paths. The expression of dynamic weight is shown in Equation (8):

(8) $$w=w_{0}\times e^{-\partial^{\prime }g(n)}$$

where $w_{0}$ represents the initial weight and its value is equal to 1, and ∂ represents the weight influence factor and its value is equal to 0.1.

To improve the search efficiency, this paper introduces dynamic weights into the future expected cost, and Equation (7) is improved to Equation (9):

(9) f(n)=g(n)+w⋅h(n)+s(n)

**Figure 3:**
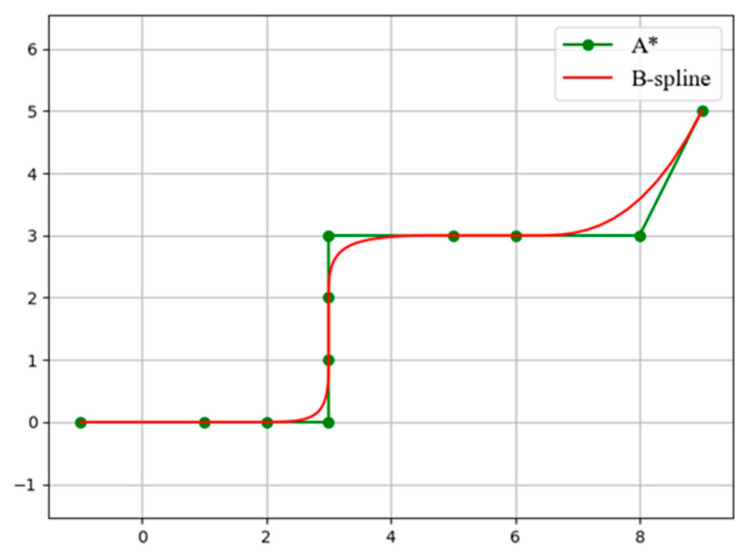
A schematic diagram of the clamped curve smoothing optimization of the A* algorithm path.

In the traditional A* algorithm, the smoothness of the agent’s motion process has not been considered, so the obtained path has inflection points and is not smooth enough, resulting in an increase in the complexity of the agent’s control. The schematic diagram of using a B-spline curve to optimize the A* path is shown in Figure 3.

**Figure 4:**
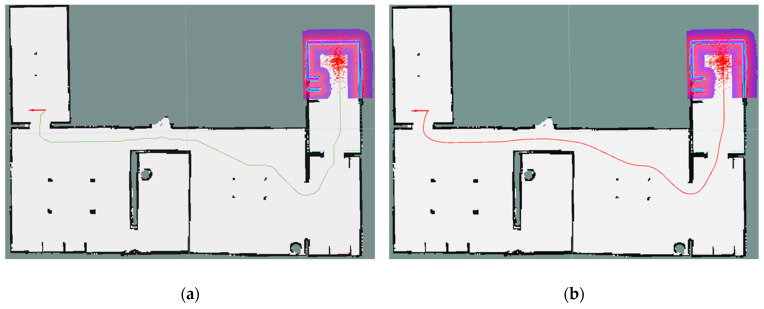
A schematic diagram comparing motion planning results. The images are labeled alphabetically as (**a**) traditional A* algorithm motion planning and (**b**) improved A* algorithm motion planning.

In order to ascertain whether the introduction of steering cost and B-spline optimization can enhance the efficacy of the algorithm, a simulation environment was constructed for the purpose of evaluating the algorithm. A comparison of the planning results of the improved A* algorithm and the traditional A* algorithm is presented in Figure 4. The red line represents the planning result of the improved A* algorithm, while the green line depicts the planning result of the traditional A* algorithm. The results in Table 2 demonstrate that the spikes at the inflection points are effectively optimized in the planning results of the improved A* algorithm.

It is worth noting that the decay factor was not chosen arbitrarily. Our choice was informed by a review of parameter settings commonly adopted in similar multi-robot path planning research and practical implementations within the ROS navigation framework. Parameters in the range of 0.05 to 0.2 are widely used in the literature for controlling weight adjustment rates in heuristic-based global planners. After preliminary tuning, a value of 0.1 was found to offer a good balance between search speed and path quality for our specific test scenarios.

During early-stage algorithm debugging and platform deployment, we conducted numerous qualitative observations (though not formally reported) under varying decay factors. Excessively large values (e.g., >0.2) led to overly aggressive convergence, compromising path optimality, while excessively small values (e.g., <0.05) significantly slowed down search efficiency. A setting of 0.1 empirically yielded stable, repeatable, and reasonably fast planning results across environments with different obstacle densities.

### 3.2. Improved TEB Algorithm

The traditional TEB algorithm has shortcomings such as a large amount of calculations when facing irregular obstacles, which leads to a reduction in local motion planning efficiency and planning failure when facing dynamic obstacles. In response to the above situation, this section has made the following improvements.

**Figure 5:**
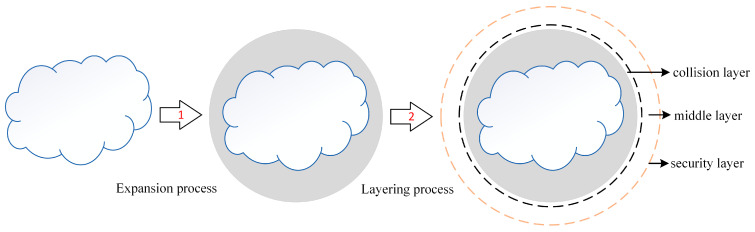
A schematic diagram of the TEB algorithm improvement.

Improvement 1: In order to improve the problem of large calculation volume caused by irregular obstacles in the TEB algorithm, resulting in reduced efficiency, all obstacles are expanded. First, an expansion model is built, the expansion model radius is initialized, and then all irregular obstacles are expanded into circles. The schematic diagram of the expansion process is shown in Figure 5.

Improvement 2: In order to improve the situation where the TEB algorithm sometimes fails to plan for dynamic obstacles, this section performs hierarchical processing on the obstacles treated by expansion and processes the obstacles into safety layers, intermediate layers, and collision layers. During the planning process, in order to avoid dynamic obstacle avoidance failures due to being too close to an obstacle, priority will be given to planning in the safety layer. If there is no planning result in the safety layer, then intermediate layer planning will be considered. If there are no planning results in either the safety layer or the intermediate layer, the planning will fail. The layered processing is shown in Figure 5.

Building upon the improvements in the A* and TEB algorithms, this section introduces the complete integrated framework for multi-robot motion planning based on the ROS Move_base architecture.

### 3.3. A Framework for the Motion Planning of Multi-Mobile Robots

The move_base framework was originally developed for the autonomous navigation of a single robot. In order to address the challenge of multi-robot motion planning [36], we propose a global and local collaborative planning algorithm based on the move_base framework. This involves extending the A* algorithm to a global planning algorithm and the TEB algorithm to a local planning algorithm, with the aim of solving complex motion planning problems in a multi-robot context.

**Figure 6:**
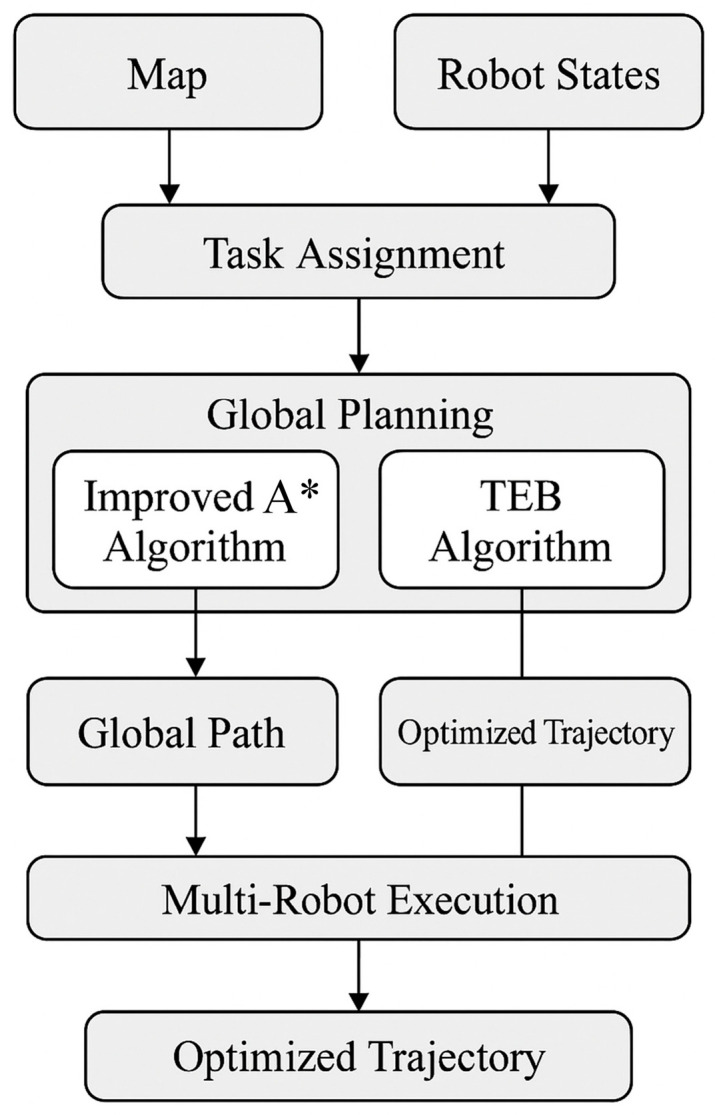
Workflow diagram of our method.

As illustrated in Figure 6, the improved A* algorithm is used to ensure the feasibility and synergy of the global paths, while the TEB algorithm is used to optimize the local paths to cope with the dynamic environment. Combining odometry and AMCL positioning techniques, the system is capable of efficient motion planning and dynamic obstacle avoidance in complex environments. The system relies on the distributed network and real-time communication mechanism of ROS to ensure fast data transfer and processing, thus enabling multiple robots to collaborate and navigate autonomously in a safe and efficient manner.

### 3.4. Conflicts and Collisions in Multi-Robot Motion Planning

**Figure 7:**
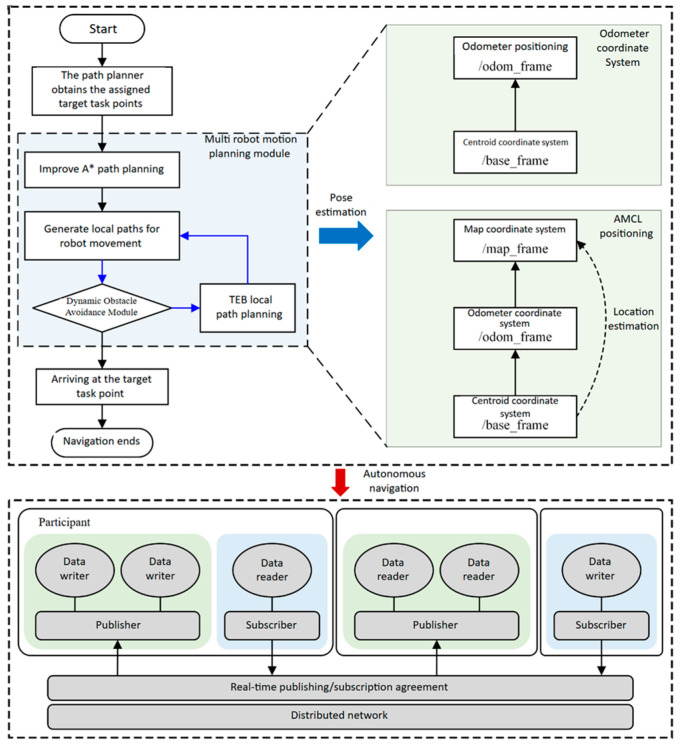
Multi-robot collaborative motion planning framework.

**Figure 8:**
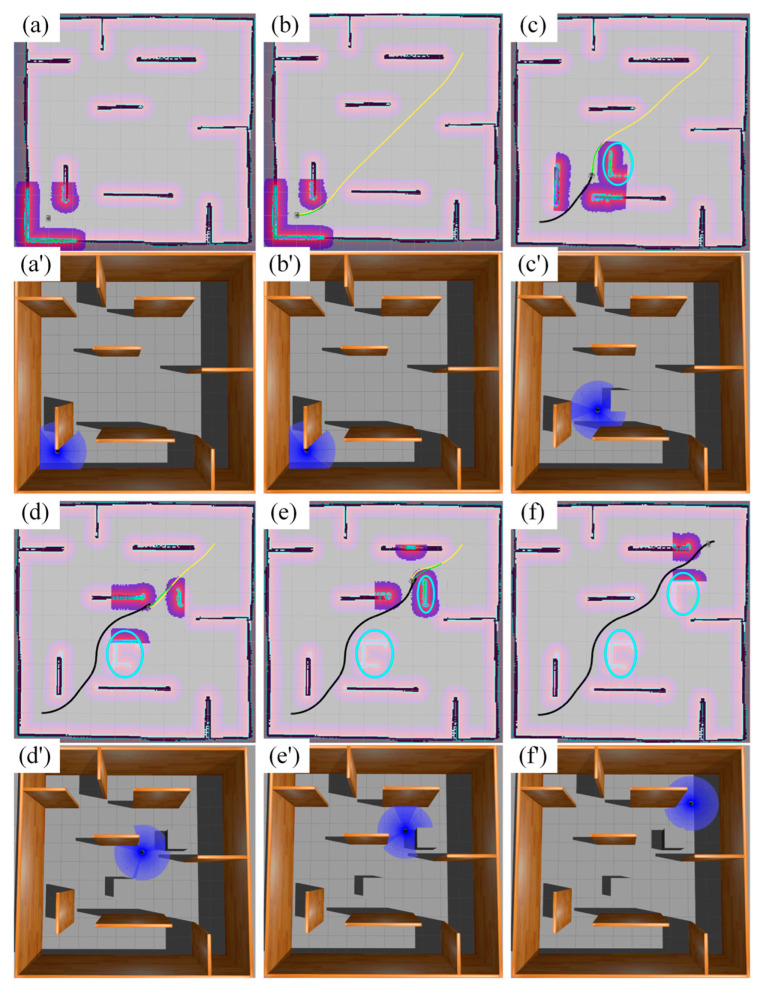
Synergistic effects of global planning and local planning. In the figure, the yellow line represents the global planning result, the green line represents the local planning result, the black line represents the robot’s driving trajectory, and the blue circle represents the unknown obstacle. From the experimental results, it can be seen that global planning and local planning are adjusted in real time during the robot’s movement. Global planning calculates a feasible path to reach the target task point, while local planning corrects and adjusts the global planning result to bypass obstacles and maintain a certain safe distance. (**a**–**f**,**a′**–**f′**) are the experimental processes at different time stages, respectively.

It is challenging to guarantee the safety and real-time performance of multi-robot motion planning. In comparison to single-robot motion planning, multi-robot motion planning must address the issue of potential conflicts between individual robot paths, as illustrated in Figure 7. Another key aspect is the ability to effectively avoid obstacles in real time. For this reason, this paper adopts an enhanced A* algorithm as the global planner to design conflict-free global guidance trajectories for multiple robots. Additionally, an improved TEB algorithm is utilized as the local planner to achieve real-time local obstacle avoidance, as shown in Figure 8.

**Figure 9:**
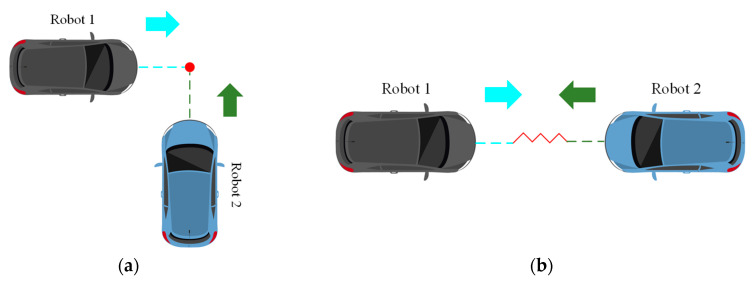
Conflict classification: (**a**) point conflict and (**b**) line conflict.

This section is dedicated to the verification of the efficacy of the proposed method for the resolution of conflicts in multi-robot motion planning, with the objective of ensuring the prevention of collisions between robots during the planning phase. The conflicts are mainly divided into two categories, one is point conflict and the other is line conflict, as shown in Figure 9. The experiments are conducted in a scenario that encompasses both point conflicts and edge conflicts, and the results are compared with those of the A* algorithm, with a view to verifying the feasibility of the proposed method.

**Figure 10:**
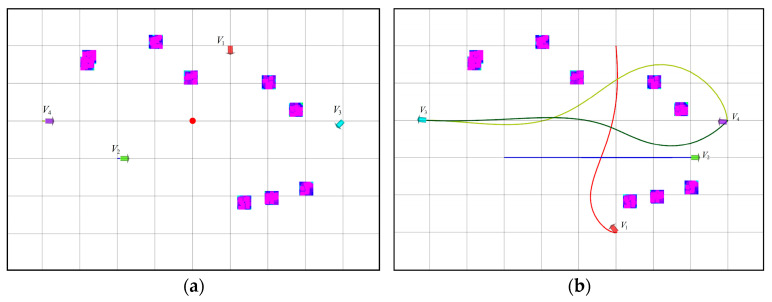
Conflict simulation: (**a**) experimental scenarios and (**b**) pathways for planning.

To evaluate the viability of our proposed methodology, we conducted a basic simulation experiment where the start coordinates and corresponding target points for four robots were randomly initialized, and the red, green, blue, and purple arrows represent the paths of robots A, B, C, and D, respectively, while the red circles indicate obstacles within the environment, as depicted in Figure 10a. As shown in Figure 10b, the final conflict-free paths were generated using the proposed method. The individual robot paths successfully avoid obstacles and remain free of conflicts.

## 4. Experimental Results and Analysis

### 4.1. Experimental Platform

In order to validate our approach, we built a multi-agent software simulation platform based on ROS, Gazebo, and Rviz, and then built a simulation experimental environment based on the software simulation platform. The algorithm studied in this article is verified through the simulation environment and actual scenarios, using a combination of three-dimensional simulation and prototype testing.

**Figure 11:**
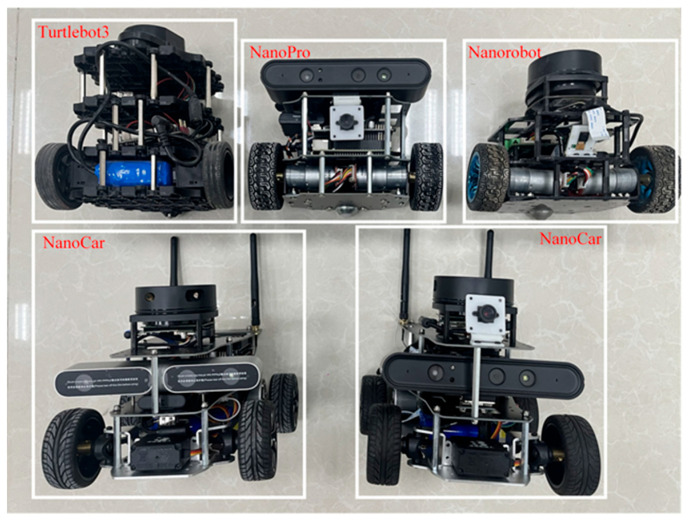
A picture of 5 mobile robots.

The hardware part of the experimental platform in this article includes one laptop and five mobile robots. The laptop is used as the host. The hardware configuration includes an Intel i7 9750H CPU processor with a main frequency of 2.6 GHz and 16 GB of memory. The five mobile robots are shown in Figure 11.

### 4.2. Prototype Verification and Analysis

**Figure 12:**
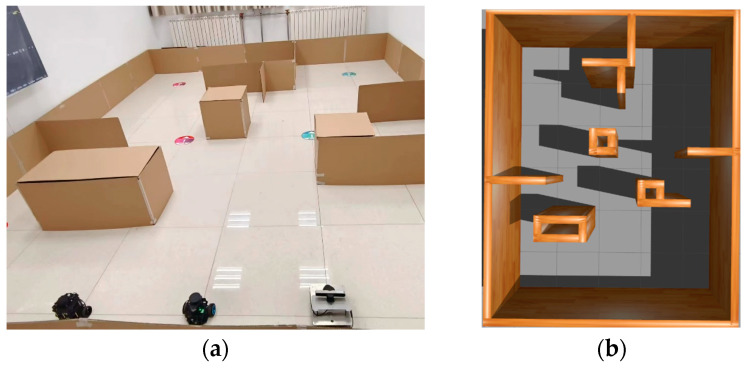
An experimental prototype scene of three mobile robots. The images are labeled alphabetically as (**a**) actual scene and (**b**) simulation scenario.

Three heterogeneous mobile robots, Turtlebot3, Nanorobot and NanoPro in Figure 12, were selected to perform prototype experiments in the scene (4.8 m × 6.0 m) shown in Figure 12a; the simulation scene in Figure 12b was built under Gazabo according to the actual situation, and the algorithm proposed in this paper is used to perform collaborative motion planning experiments. The TurtleBot3 is manufactured by ROBOTIS Co., Ltd. in Daejeon, Republic of Korea; both the Nanorobot and NanoPro are sourced from manufacturers based in China.

All robots returned to their initial position after passing the navigation point. The experimental process of the simulation scenario built based on the actual scene is shown in Figure 10. The three colored lines of blue, green, and red in the figure represent the running trajectories of the three mobile robots, respectively.

**Figure 13:**
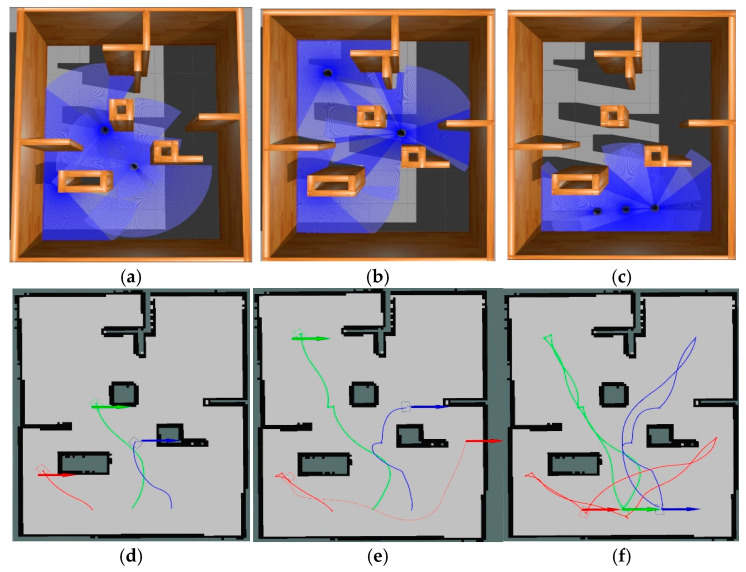
This is the simulation experiment process diagram of 3 mobile robots. Different colors represent the three robots. The images are labeled alphabetically as (**a**) process 1 under Gazebo, (**b**) process 2 under Gazebo, (**c**) process 3 under Gazebo, (**d**) process 1 under Rviz, (**e**) process 2 under Rviz, and (**f**) process 3 under Rviz.

The experiment of the mobile robots uses the total time to complete the task and the total driving distance of the mobile robot as evaluation indicators. In order to avoid the impact of accidental errors, five experiments were conducted in simulation scenarios and actual scenarios. Figure 12 and Figure 13 show the statistics of the total time and total distance traveled by the mobile robots in the simulation scenario and the actual scenario of the five mobile robots, respectively.

**Figure 14:**
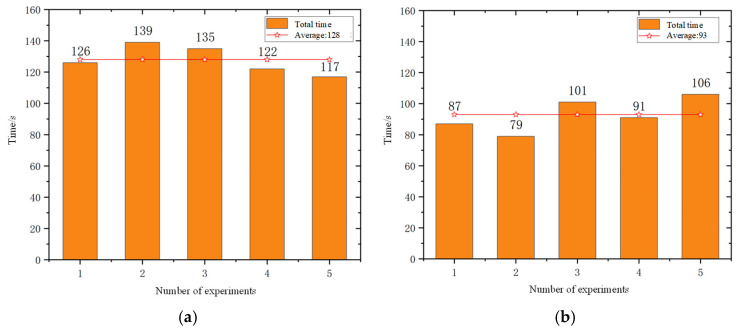
Statistics of the total time to complete tasks: (**a**) simulation scenario and (**b**) actual scene.

**Figure 15:**
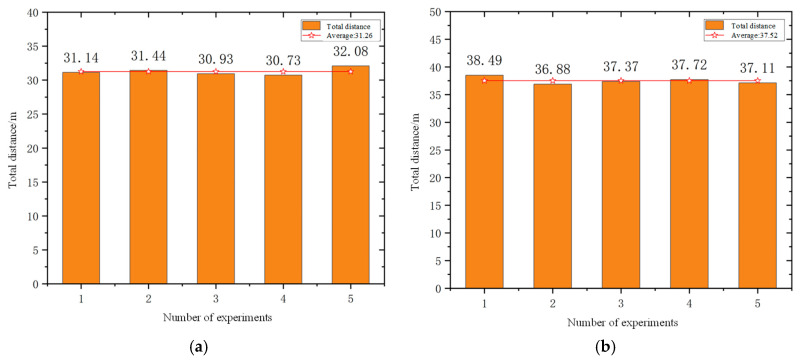
Statistics of the total distance to complete tasks: (**a**) simulation scenario and (**b**) actual scene.

According to the statistical results presented in Figure 14 and Figure 15, the average total task completion time in the real-world scenario was reduced by 27.3% compared to that in the simulation environment, while the average total travel distance increased by 20.1%. This discrepancy can be attributed to several factors. In the simulation environment, the real-time navigation of multiple mobile robots imposes high computational demands on the processor, resulting in prolonged simulation time due to limited processing speed. In contrast, in the real-world scenario, the travel distance is measured based on odometry data. The heterogeneous mobile robots operating in the actual environment are subject to external disturbances such as sensor noise, odometry drift, and communication latency. Additionally, inconsistencies in motion control speed across different prototype robots further contribute to the increase in travel distance. Moreover, the path planning results in the simulation are more idealized, as they consider fewer practical constraints. In real-world conditions, environmental factors such as terrain irregularities and obstacle distribution may lead to the generation of more optimized paths, thereby reducing the total task completion time. Consequently, there exists a notable performance gap between real-world and simulated scenarios.

**Figure 16:**
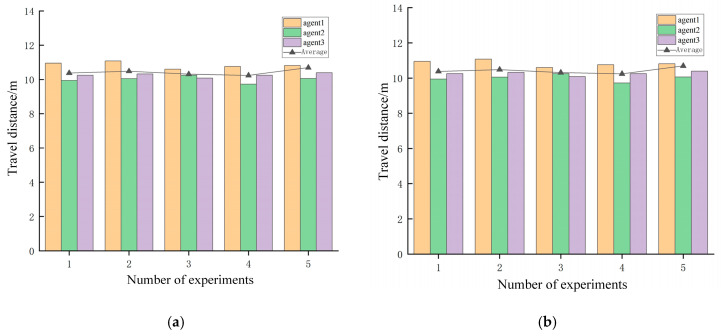
Travel distance of each of the three mobile robots: (**a**) simulated scenario and (**b**) actual scenario.

According to the statistical results shown in Figure 16, the deviation between each mobile robot’s travel distance and the average travel distance remained within an absolute value of 1 in both the simulation and real-world environments. Furthermore, for each experimental group, the difference between the maximum and minimum travel distances among the mobile robots was within a value of 2. These results collectively demonstrate the rationality and effectiveness of the task allocation strategy.

As the number of robots in the system increases, the complexity of multi-robot motion planning also rises. In order to validate the feasibility of the method in a greater number of multi-robot systems, we conducted additional experiments involving five heterogeneous robots in the scenario shown in Figure 16a (4.8 m × 6.0 m), and the simulation scenario shown in Figure 16b was built under Gazabo according to the actual situation.

The experimental design is for five mobile robots to complete five target tasks, as shown in Table 3. Notably, two of the robots were tasked with returning to their starting points after completing their assigned tasks. The details of the robots and target navigation points are summarized in Table 4. The mobile robot P1 navigates to the target point P1 and then returns to the starting point, the mobile robot R2 navigates to P2, the mobile robot R3 navigates to P3, the mobile robot P4 navigates to the target point P4 and then returns to the starting point, and the mobile robot R5 navigates to P5.

**Figure 17:**
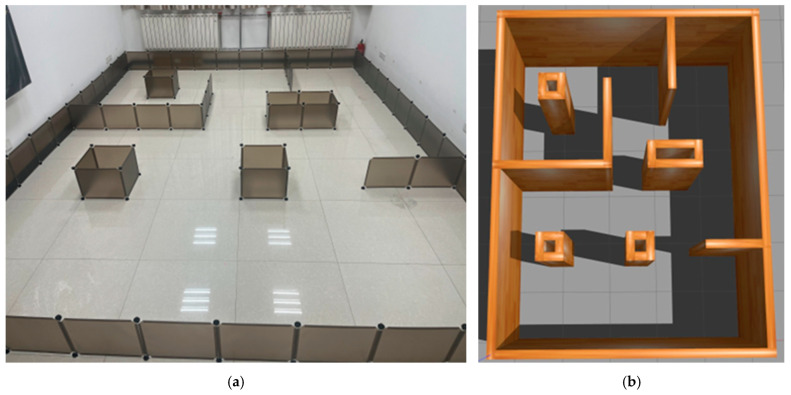
An experimental prototype scene of five mobile robots. The images are labeled alphabetically as (**a**) actual scene and (**b**) simulation scenario.

**Figure 18:**
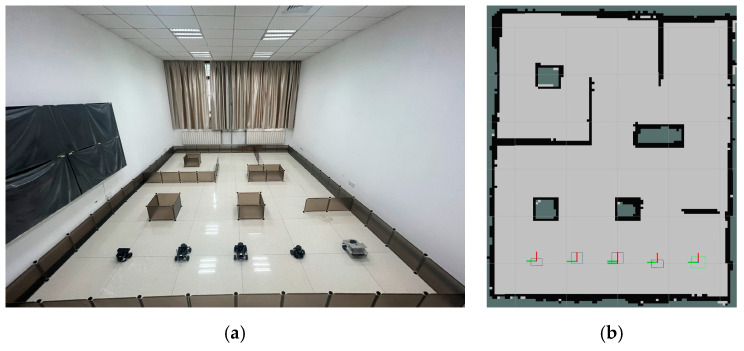
A schematic diagram of the actual starting positions of the five mobile robot prototype experiments. The images are labeled alphabetically as (**a**) the prototype starting position of the actual scene and (**b**) the experimental prototype map under Rviz.

**Figure 19:**
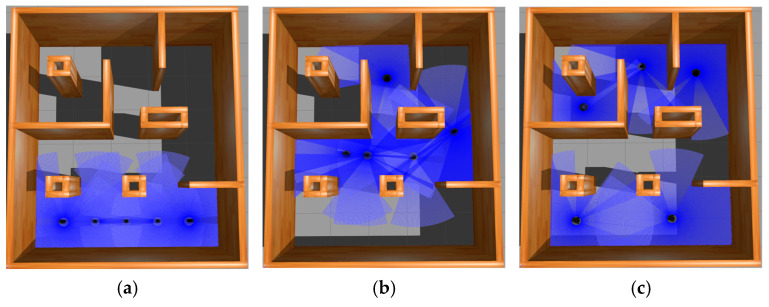

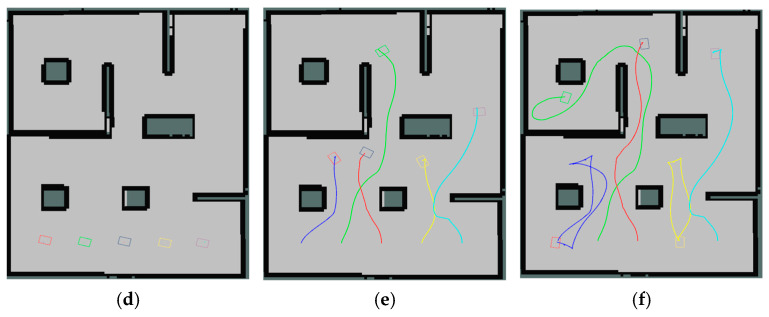
This is the simulation experiment process diagram of 5 mobile robots. The images are labeled alphabetically as (**a**) process 1 under Gazebo, (**b**) process 2 under Gazebo, (**c**) process 3 under Gazebo, (**d**) process 1 under Rviz, (**e**) process 2 under Rviz, and (**f**) process 3 under Rviz.

The experimental prototype process is shown in Figure 17 and Figure 18. The boxes in Figure 18a represent the initial positions of the five mobile robots. The experimental process of the simulation scenario built based on the actual scene is shown in Figure 19. The five colored lines of dark blue, green, red, yellow, and light blue in the figure represent the running trajectories of the five mobile robots, respectively.

**Figure 20:**
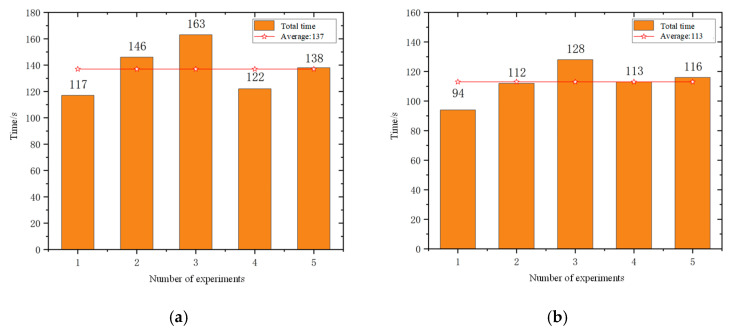
Statistics of the total time to complete tasks: (**a**) simulation scenario and (**b**) actual scene.

**Figure 21:**
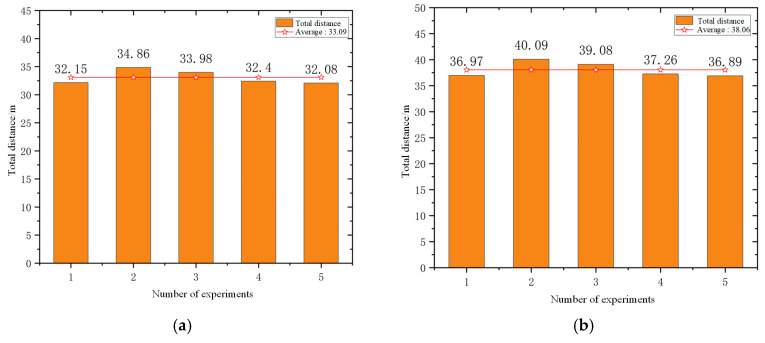
Statistics of the total distance to complete tasks: (**a**) simulation scenario and (**b**) actual scene.

The experiment of five mobile robots uses the total time to complete the task and the total driving distance of the mobile robot as evaluation indicators. In order to avoid the impact of accidental errors, five experiments were conducted in simulation scenarios and actual scenarios. Figure 20 and Figure 21 show the statistics of the total time and total distance traveled by the mobile robots in the simulation scenario and the actual scenario of the five mobile robots, respectively.

The experimental results show that all robots completed the navigation tasks. From the statistical results in Figure 20 and Figure 21, it can be seen that the average total time to complete the task in the actual scenario is 17.5% lower than that in the simulation scenario, and the average total driving distance in the actual scenario is 15% higher than that in the simulation scenario. Through analysis, it can be seen that the real-time navigation of multiple mobile robots in the simulation environment requires high processor performance. The processing speed of the processor causes the simulation time to be too long. However, the distance of the mobile robots in the actual environment is calculated through the odometer. In actual scenarios, heterogeneous mobile robots have the phenomenon of odometer drift and communication delay, and in the prototype experiment, the motion control speed of each mobile robot is different, and the road conditions in the simulation environment are relatively ideal, so there is a gap between the actual scenario and simulation scenes.

**Figure 22:**
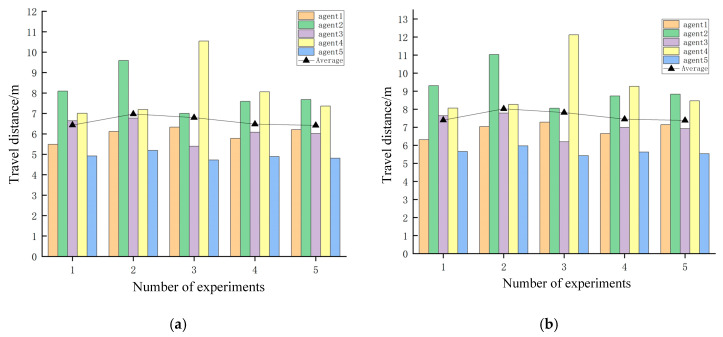
Travel distance of each of the five mobile robots: (**a**) simulation scenario and (**b**) actual scene.

According to the statistical results presented in Figure 22, a relatively large deviation was observed between each mobile robot’s travel distance and the average value in both the simulation and real-world environments. This discrepancy can be attributed to two primary factors. First, among the five mobile robots, two were required to return to their starting positions after completing their assigned tasks, resulting in an increase in total travel distance. Second, the path planning algorithm employs a heuristic function, which introduces a degree of randomness, leading to variability across experimental trials. Nevertheless, all statistical results remain within an acceptable and reasonable range.

**Figure 23:**
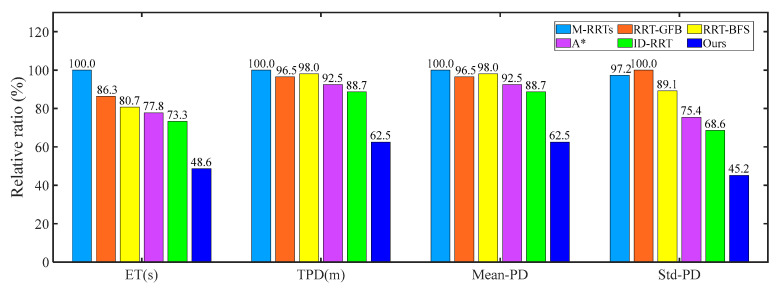
The relative ratio of the contrast experimental results.

As illustrated in Figure 23, an analysis was conducted between our method and three existing approaches: The M-RRTs [37], RRT-GFB [38], RRT-BFS [39], A* [40], and ID-RRT algorithms [41]. This comparison aimed to provide a comprehensive evaluation of our method’s performance, as shown in Table 5. The corresponding experiments were conducted in each of the five real-world environments for robot motion planning. Furthermore, the experiment utilizes the total time to complete the task and the total driving distance of the mobile robot as evaluation criteria. For the total distance, the method with the longest distance was used as the 100% benchmark, while for the running time, the method with the longest time was set as the 100% benchmark. The analysis results show that our proposed method outperforms the other four methods; our method reduces the total distance by up to 29.5% and the time by up to 30% compared to the other methods.

## 5. Conclusions

This paper presented A-TEB*, a unified motion planning framework that combines an enhanced A* global planner with the TEB local planner to tackle multi-robot path planning challenges. The approach effectively resolves inter-robot path conflicts while improving real-time dynamic obstacle avoidance by leveraging the complementary strengths of global and local planning. Simulation and real-world experiments with up to five heterogeneous robots demonstrated significant performance gains: the improved A* produced shorter and smoother global paths (about 5% reduction in path length and two-thirds fewer turning maneuvers compared to the standard A*), which in turn lowered travel time and improved efficiency. Meanwhile, the modified TEB algorithm enabled responsive, collision-free obstacle avoidance, ensuring that all robots safely reached their goals without conflict. Overall, the proposed A*-TEB framework outperformed several baseline methods, reducing total travel distance by up to 27.7% and completion time by about 15% in comparative trials. These results underscore the framework’s practical significance as a robust solution for multi-robot systems that are ready for deployment in real-world applications such as automated warehouses, coordinated exploration, and other domains requiring efficient cooperative navigation.

In future work, we aim to extend the A*-TEB framework to larger and more heterogeneous robot teams to further assess scalability and coordination in complex scenarios. Another promising direction is to integrate learning-based planners (e.g., reinforcement learning or neural motion planners) with our framework, allowing robots to adaptively improve their planning strategy based on experience and environmental feedback. Additionally, we plan to address non-holonomic kinematic constraints (such as those in car-like robots or aerial drones) by refining the planner to consider motion models with limited turning radii, thereby broadening the framework’s applicability to a wider range of robotic platforms. These enhancements will further improve the robustness and versatility of the proposed approach, paving the way for more scalable, intelligent, and reliable multi-robot motion planning in real-world environments.

## Figures and Tables

**Table 1:** The comparative analysis of various A* improvements and TEB variants.

Algorithm	Reference	Improved Method	Disadvantages	Advantages
A*	Ref. [11]	improved only	limited scalability	effectively resolves the planning conflicts among multiple robots
	Ref. [12]	improved only	falling into local optima	collision-free, smooth, and near-optimal paths
	Ref. [13]	improved only	significant computational cost	fewer turning points
	Ref. [14]	improved only	lacks adaptability to varying scenarios	prejudgment planning
	Ref. [15]	combined others	lacks adaptability to highly dynamic environments	maintain safe distances from obstacles
	Ref. [16]	combined others	tends to generate locally suboptimal paths	fewer path segments and turning points
TEB	Ref. [17]	combined others	algorithm’s performance heavily depends on grid resolution	improved search efficiency, shorter paths, and better obstacle avoidance
	Ref. [26]	variants	relies on heuristic point selection	enables dynamic, kinematically feasible path planning
	Ref. [27]	variants	does not incorporate robot kinematics or real-time egocentric perception	deterministic and globally consistent path planning

**Table 2:** Comparison of results.

	Improved A* (∂ = 0.2)	Improved A* (∂ = 0.05)	Improved A* (∂ = 0.1)	A*
Average path length/m	23.01	21.44	19.29	20.35
Average completion time/s	76.80	82.17	74.3	83.95
Average number of inflection points	3	3	3	9
Average number of nodes	129.52	170.59	117.2	208.4

**Table 3:** Location information.

	R1	R2	R3	R4	R5	P1	P2	P3	P4	P5
X-axis/m	0.8	1.6	2.4	3.2	4.0	1.0	1.0	2.5	3.0	4.0
Y-axis/m	0.8	0.8	0.8	0.8	0.8	2.5	4.0	5.2	2.5	5.0

**Table 4:** Robot model and related technical parameters.

Robot Model	Turtlebot3	NanoPro	Nanorobot	NanoCar
Body diameter or body size/mm	155	160	160	180 × 160
Line speed/m	0.25	0.25	0.25	0.25
Angular velocity/(rad/s)	0.15	0.15	0.15	0.15
Radar scanning radius/m	5	5	5	5
Position measurement frequency/Hz	100	100	100	100
Position accuracy/mm	0.5~1	0.5~1	0.5~1	0.5~1
Attitude accuracy/(°)	1~2	1~2	1~2	1~2

**Table 5:** The relative ratio of the contrast experimental results.

Algorithm	ET (s)	TPD (m)	Mean-PD	Std-PD
M-RRTs	104.19	37.43	12.4767	6.7269
RRT-GFB	89.96	36.13	12.0433	6.9187
RRT-BFS	84.11	36.69	12.2300	6.1677
A*	81.05	34.61	11.5367	5.2200
ID-RRT	76.34	33.19	11.0633	4.7454
**Ours**	**50.66**	**23.41**	**7.8033**	**3.1270**

Note: ET is the execution time, and TPD is the total path distance.

## Data Availability

The original contributions presented in this study are included in the article. Further inquiries can be directed to the corresponding author.
